# KCC2 phosphorylation dynamics relate to oligomerization during development and after spinal cord injury

**DOI:** 10.3389/fnmol.2026.1745037

**Published:** 2026-02-04

**Authors:** Sylvie Liabeuf, Cecile Brocard, Jacques-Olivier Coq, Laurent Vinay, Frederic Brocard

**Affiliations:** Institut de Neurosciences de la Timone, UMR 7289, Aix-Marseille Université and Centre National de la Recherche Scientifique (CNRS), Marseille, France

**Keywords:** inhibitory neurotransmission, chloride homeostasis, KCC2, oligomerization, phosphorylation, spinal cord, spinal cord injury

## Abstract

Effective synaptic inhibition relies on KCC2, a K^+^-Cl^−^ cotransporter that forms oligomers to extrude chloride. However, how phosphorylation regulates oligomerization remains unclear. To address this, we studied the phosphorylation of KCC2 in the lumbar spinal cord of neonatal rats by subcellular fractionation and phospho-specific western blotting across three paradigms with distinct expression profiles: developmental upregulation, spinal cord injury (SCI)-induced downregulation, and pharmacological rescue with the 5-HT_2_A/_2_C agonist DOI. KCC2 moved from cytoplasm to membrane during development, exhibiting increased intracellular phosphorylation at serine, threonine, and tyrosine residues and decreased membrane tyrosine phosphorylation as a result of the oligomer formation. SCI caused a shift in this ratio in favor of lower oligomers, higher membrane tyrosine phosphorylation and lower intracellular threonine phosphorylation. DOI partially reversed these deficits, reinforcing oligomerization and adapting phosphorylation to a developmental profile. In sum, this study identifies tyrosine dephosphorylation at the membrane as a reliable correlate of oligomerization and offers a unified quantitative model for the regulation of KCC2 in development, after injury, and upon pharmacological rescue.

## Introduction

In the immature nervous system, GABA and glycine are not inhibitory transmitters, but they depolarize neurons and in some cases even excite neurons (Mandler et al., 1990; Wu et al., 1992; Gao and Ziskind-Conhaim, 1995; Ziskind-Conhaim, 1998; Czarnecki et al., 2014; Branchereau et al., 2016). This excitatory effect is mediated through, at least, the cotransporter NKCC1, which, by loading immature neurons with chloride, shifts the reversal potential towards more depolarized values (Rohrbough and Spitzer, 1996; Ben-Ari et al., 2007). As a consequence, GABA- or glycine-induced depolarizations trigger Ca^2+^ entry, supporting developmental processes such as neurogenesis and synaptogenesis (Reichling et al., 1994; Wang et al., 1994; Serafini et al., 1995; Represa and Ben-Ari, 2005; Momose-Sato et al., 2007; Bortone and Polleux, 2009).

This situation is reversed as the neurons mature. KCC2 levels gradually increase, lowering intracellular chloride and converting GABAergic and glycinergic signaling from depolarizing to hyperpolarizing (Plotkin et al., 1997; Rivera et al., 1999; Hubner et al., 2001; Ueno et al., 2002; Payne et al., 2003; Ben-Ari et al., 2007; Blaesse et al., 2009; Kaila et al., 2014; Watanabe and Fukuda, 2015; Doyon et al., 2016). In the spinal cord, this transition occurs in rat lumbar motoneurons shortly after birth, when KCC2 expression is upregulated (Jean-Xavier et al., 2006; Stil et al., 2009). This occurs at the same time as descending brainstem serotonergic projections have reached the spinal cord (Bregman, 1987; Rajaofetra et al., 1989; Lakke, 1997; Brocard et al., 1999; Vinay et al., 2002), that appear to promote KCC2 upregulation, and as a result the maturation of inhibitory circuits (Pflieger et al., 2002).

In parallel with the developmental regulation of KCC2, a large body of evidence has demonstrated a direct relationship between KCC2 dysregulation and spinal cord dysfunction. After spinal cord injury (SCI), KCC2 is downregulated in particular at motoneuronal membranes, depolarizing the inhibitory reversal potential (E*_Cl_* /E*_IPSP_*), and reducing inhibitory synaptic transmission, which results in hyperreflexia and spasticity (Jean-Xavier et al., 2006; Boulenguez et al., 2010; Cote et al., 2014). More broadly, KCC2 downregulation and the resulting loss of chloride homeostasis has also been involved in neuropathic pain signaling within the dorsal horn circuitry (Coull et al., 2003; Mapplebeck et al., 2019). Conversely, those that enhance KCC2 function, such as 5-HT_2A/2C_ receptor activation (e.g., DOI), will hyperpolarize E*_Cl_*/E*_IPSP_*, increase KCC2 membrane expression, strengthen inhibition, and alleviate symptoms associated with SCI, suggesting that KCC2 is a viable therapeutic target post-injury (Bos et al., 2013; Liabeuf et al., 2017; Sanchez-Brualla et al., 2018; Beverungen et al., 2020; Malloy and Cote, 2024).

Besides transcriptional regulation, KCC2 function is also regulated by post-translational modifications. Its trafficking, surface expression, and transport efficiency are all influenced by phosphorylation of its C-terminal residues (Russell, 2000; Blaesse et al., 2009; Kahle and Delpire, 2016). Some studies even argue that phosphorylation could regulate KCC2 oligomerization, which is usually assumed to be required for proper transport (Chamma et al., 2012; Kahle et al., 2013; Medina et al., 2014; Come et al., 2019). Still, the functional consequences of phosphorylation are still to be completely elucidated: some studies point to stabilization at the surface, whereas others implicate specific sites that promote internalization or reduce transport capacity (Strange et al., 2000; Kelsch et al., 2001; Bergeron et al., 2006; Wake et al., 2007; Watanabe et al., 2009; Lee et al., 2010; Chamma et al., 2012; Kahle et al., 2013; Medina et al., 2014). These discrepancies are probably due to variations in experimental design, developmental stage, or the particular residues analysed.

In this context, the present work is a continuation of previous functional studies, with the goal of providing a unified biochemical explanation of KCC2 dynamics. We thus used the same experimental paradigm and developmental time window to assess the phosphorylation status of KCC2 under three conditions that can be considered paradigmatic: (1) its upregulation during postnatal development, (2) its downregulation following SCI, and (3) its pharmacological restoration by the 5-HT_2_A/_2_C agonist DOI. In particular, we limited our scope to the neonatal rat lumbar spinal cord at one specific early developmental age (P7) and employed subcellular fractionation, pan-phospho-specific immunoblotting and multivariate analysis. By using this method, we were able to identify common phosphorylation events correlated with oligomerization.

## Materials and methods

### Experimental model and subject details

#### Animals

Neonatal Wistar rats (Charles River, France) of both sexes, aged postnatal day 0 (P0) to P7, were used in this study. Animals were housed under a 12-h light/dark cycle with ad libitum access to water and food, at 21–24 °C and 40–60% relative humidity. All procedures complied with French regulations (Décret 2010–118) and were approved by the ethics committee (Comité d’Ethique en expérimentation animale, CEEA-071, Nb B1301404, authorization Nb 2,018,110,819,197,361).

### Method details

#### Surgical procedures

Surgeries were performed under hypothermia anesthesia on the day of birth (P0). Prior to surgery, pups received preventive analgesia with buprenorphine (0.05 mg/kg, s.c.) and antisepsis (povidone-iodine 10%). A laminectomy was performed, and the spinal cord was transected at T8-T10, removing one or two segments as described (Jean-Xavier et al., 2006; Sadlaoud et al., 2010). The lesion cavity was filled with sterile absorbable hemostat Surgicoll, amoxicillin was applied locally and skin incisions were sutured with fine thread and covered with Steri-Strips (3 M Health Care, St. Paul, MN). After wound closure, pups were rewarmed on a heating pad inside a humidified chamber containing nesting material with the dam’s scent, and returned to the litter once spontaneous movement, pink coloration and regular breathing were restored. Pups were inspected twice daily for weight gain, skin integrity, posture and activity. Humane endpoints included failure to recover normal movement within 45 min, > 10% weight loss at 24 h, persistent signs of distress, or suture dehiscence, in which case animals were euthanized. For tissue collection, pups (≤ P7) were euthanized by rapid decapitation, in accordance with ethical approval.

#### DOI treatment

From P4 to P7, SCI rats received daily intraperitoneal injections of 0.15 mg/kg 2,5-dimethoxy-4-iodoamphetamine hydrochloride (DOI; Sigma-Aldrich) diluted in 50 μL NaCl, a 5-HT_2_A/_2_C receptor agonist that crosses the blood-spinal cord barrier (Kim et al., 1999; Norreel et al., 2003; Sadlaoud et al., 2010). Control SCI rats (n = 6) received 50 μL NaCl daily (vehicle). Injections were administered using a 1 mL syringe with a 26-gauge needle.

#### Subcellular fractionation and immunoprecipitation

Spinal cords caudal to the lesion site (T8) were rapidly dissected from animals at P0 and P7. For each sample, four spinal cords were pooled at P0 and two at P7. For total protein extraction (TF), tissues were homogenized in cold lysis buffer A (1% Igepal CA-630, 0.1% SDS, 10 mM sodium vanadate, 10 mM sodium fluoride, 10 mM sodium pyrophosphate, 10 mM iodoacetamide, supplemented with protease inhibitor cocktail [Complete Mini, Roche Diagnostics]) and centrifuged at 18,000 g for 30 min at 4 °C. For subcellular fractionation, tissues were homogenized in lysis buffer B (320 mM sucrose in 50 mM Tris–HCl, pH 7.5, with 10 mM sodium vanadate, 10 mM sodium fluoride, 10 mM sodium pyrophosphate, 10 mM iodoacetamide, supplemented with protease inhibitor cocktail) and centrifuged at 7,000 g for 5 min, then 18,000 g for 70 min at 4 °C to separate the intracellular (supernatant) and membrane (pellet) fractions. The membrane pellet was then resuspended in lysis buffer A.

Protein concentration in fractions was determined using DC protein assay (Bio-rad). The same amount of protein was analyzed by western blot (30 μg) or incubated (400 μg) with 2 μg of affinity-purified rabbit anti-KCC2 polyclonal antibody (Merck-Millipore, Billerica, MA, USA) for 4 h at 4 °C, and the protein-antibody complex was precipitated with PureProteome Protein G Magnetic Beads (Merck-Millipore). After washes in lysis buffer, beads were resuspended in an equal volume of SDS sample buffer and heated at 70 °C for 7 min. Equal volumes of samples were loaded per lane, and unloaded volumes were verified to ensure consistency.

#### Western blots

Samples were separated in 6% SDS PAGE and transferred onto a PVDF membrane. Once blocked in Tris buffer saline + 5% non-fat dry milk, membranes were probed overnight at 4 °C with primary antibodies (see table below). ImmunoPure goat horseradish peroxidase (HRP)-conjugated secondary antibodies were used for detection in a chemiluminescent system (Thermo Scientific, Waltham, MA, USA). Non-saturated signal intensity was measured with the image analysis software Quantity-One or Image Lab (Bio-Rad, Hercules, CA, USA). Background was subtracted using a rolling disk.

The following primary antibodies were used in this study: KCC2 antibody (07–432, Merck Millipore, 1/1000 dilution), phosphoserine and phosphotreonine antibodies (61–8,100 and 71–8,200, respectively, Zymed laboratories, 1/1000 dilution), phosphoTyrosine antibody (4G10, 05–321, DHSB, 1/500 dilution), Sumo1 antibody (SUMO-1 21C7, DHSB, 1/500), Nav channel *α* subunit antibody (clone K58/35, S8809, Sigma Aldrich, 1/500 dilution), and *β* III tubulin antibody (T2200, Sigma Aldrich, 1/50000 dilution).

#### Enzymatic deglycosylation

To assess the glycosylation status of KCC2, deglycosylation was performed using Endoglycosidase H (EndoH) or Peptide-N-Glycosidase F (PNGase F) (New England Biolabs). Eighty-five micrograms of protein were incubated for 2 min at 70 °C in denaturing buffer (0.5% SDS, 40 mM DTT), then for 1 h at 37 °C in reaction buffer supplemented with either 2,500 units of EndoH (in 50 mM sodium phosphate, pH 5.5) or 1,250 units of PNGase F (in 50 mM sodium phosphate, pH 7.5, with 1% NP-40).

#### *In silico* prediction of sumoylation sites

Potential sumoylation sites in rat KCC2b were predicted using three online tools: GPS-SUMO, SUMOplot (Abgent), and PCI-SUMO. These algorithms identify consensus sumoylation motifs of the form *Ψ*-K-x-D/E, where Ψ represents a large hydrophobic residue (typically I, L, V, or M), K is the lysine residue targeted for SUMO conjugation, x is any amino acid, and D/E represents aspartic acid or glutamic acid.

### Quantification and statistical analysis

#### Quantification of Western blot signals

Relative KCC2 expression was calculated by measuring band intensity and normalizing to the appropriate control condition for each experimental comparison: P0 for developmental studies (P0 vs. P7), intact P7 for injury studies (intact vs. SCI), and vehicle-treated SCI for pharmacological rescue studies (SCI vs. SCI + DOI).

For phosphorylation analysis, phosphorylation levels were calculated as the ratio of signal intensity obtained with phospho-specific antibodies (anti-phosphoserine, anti-phosphothreonine, or anti-phosphotyrosine) to the signal intensity of the total KCC2 monomeric form (130–140 kDa) within the same subcellular fraction. Relative phosphorylation levels were then normalized to the corresponding control group as described above. It is important to note that the analysis relied on pan-specific antibodies targeting total phosphotyrosine, phosphoserine, or phosphothreonine residues. Consequently, the signals quantified here reflect the global phosphorylation state of the KCC2 protein and do not allow for the identification of specific phosphorylated amino acid residues.

#### KCC2 subcellular distribution metrics

KCC2 subcellular localization was quantified using the membrane-to-intracellular ratio (Mb/Int), where Mb represents total membrane KCC2 (monomers + oligomers) and Int represents intracellular KCC2. A ratio > 1 indicates preferential membrane localization. The oligomeric fraction of membrane KCC2 was calculated as the proportion of oligomeric KCC2 (>250 kDa) relative to the total membrane pool of KCC2 (monomeric + oligomeric forms).

#### Principal components analysis (PCA)

PCA was performed using R software (version 4.1.0) with the FactoMineR and psych packages. The analysis encompassed 12 variables indicative of KCC2 expression and subcellular distribution: total KCC2 (Tot), intracellular monomer (Int-Mono), total membrane KCC2 (Mb), membrane monomer (Mb-Mono), oligomers (Oligo), membrane-to-intracellular ratio (Mb/Int), and oligomer-to-membrane ratio (Oligo/Mb). Additionally, phosphorylation at tyrosine, serine, and threonine residues in both membrane and intracellular fractions was quantified (pTyr-Mb, pSer-Mb, pThr-Mb, pTyr-Int, pSer-Int, pThr-Int), resulting in 12 phosphorylation variables. According to Kaiser’s criterion (eigenvalue > 1), the first two principal components were retained, accounting for approximately 60–77% of the total variance depending on the comparison. We calculated the quality of representation (Cos^2^ values) and the contribution of variables to each dimension. Statistical significance of group separation along principal components was assessed using V-tests (|*V*| > 2 indicates *p <* 0.05), and differences between group barycenters were tested using the F-test and Student’s *t*-test following confirmation of normality with the Shapiro–Wilk test.

#### Statistical analyses

For pairwise comparisons between experimental groups (P0 vs. P7, intact vs. SCI, SCI vs. SCI + DOI), statistical significance was determined using two-tailed non-parametric Mann–Whitney U tests (GraphPad Prism version 9.0, GraphPad Software, San Diego, CA, USA). A significance threshold of *p <* 0.05 was used for all comparisons. Data are represented as the median with interquartile range unless otherwise indicated in figure legends. Sample sizes (n) represent biological replicates (individual animals or pooled samples as specified) and are indicated in each figure legend.

## Results

To investigate how phosphorylation influences KCC2 regulation, we quantified its expression, phosphorylation state, and oligomerization in neonatal rats. Western blot analyses distinguished the inactive monomeric form from the functionally active oligomeric assemblies (Blaesse et al., 2006; Mahadevan et al., 2014), and we separated total, membrane, and intracellular fractions to assess both spatial and temporal regulation.

### Developmental dynamics of KCC2 distribution and phosphorylation

**Figure 1:**
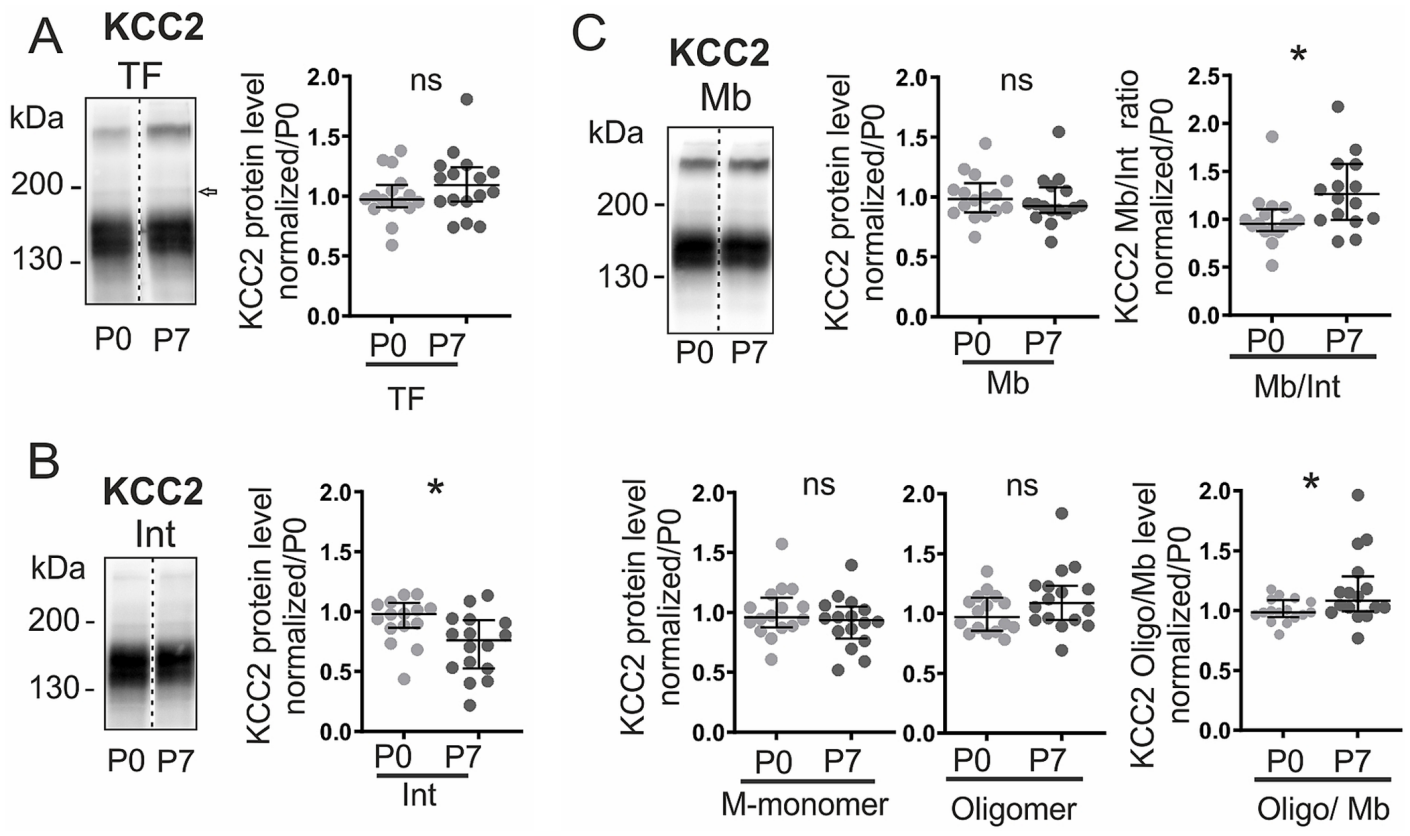
Developmental dynamics of KCC2 distribution and expression. KCC2 expression at postnatal days 0 (P0) and 7 (P7). **(A)** Total KCC2 immunoblot and quantification. **(B)** Intracellular fraction. **(C)** Membrane fraction: total KCC2 (*top middle*), membrane-to-intracellular ratio (Mb/Int, *top right*), monomers (*bottom left*), oligomers (*bottom middle*), and oligomer-to-membrane ratio (*bottom right*). All values normalized to P0. The arrow in (**A)** indicates the presence of a minor high-molecular-weight KCC2 species (~180 kDa) in the total lysate, corresponding to the tyrosine-phosphorylated pool described in Figure 2. Each dot consists of pooled spinal cords (four at P0, two at P7). *p < 0.05; *n* = 16 per group; n represents pooled biological samples. Mann–Whitney test. Data are represented as median and interquartile range. See also Supplementary Figure S1.

During the first postnatal week, KCC2 was mainly detected as monomers (130–140 kDa) in both membrane and intracellular fractions, while oligomers (> 250 kDa) were confined to the plasma membrane (Supplementary Figure S1a). This distribution pattern, confirmed with compartment-specific markers (*β*-tubulin III for intracellular, Nav *α*-subunit for membrane; Supplementary Figure S1b), remained stable from birth (P0) to postnatal day 7 (P7) (Supplementary Figure S1c). Total KCC2 levels did not change significantly between P0 and P7 (Figure 1A). However, the subcellular distribution shifted: intracellular KCC2 declined by 22% (*p <* 0.05; Figure 1B), while membrane levels remained stable (Figure 1C), resulting in a 31% increase in the membrane-to-intracellular ratio (Mb/Int; *p <* 0.05; Figure 1B). Within the membrane fraction, although the absolute amount of membrane monomers and oligomers remained unchanged (Figure 1B), the oligomer proportion rose by 10% (*p <* 0.05; Figure 1B), indicating a developmental shift toward the functionally active oligomeric form. This redistribution occurred in parallel with the acquisition of mature glycosylation (EndoH resistance, PNGase F sensitivity; Supplementary Figure S1c).

**Figure 2:**
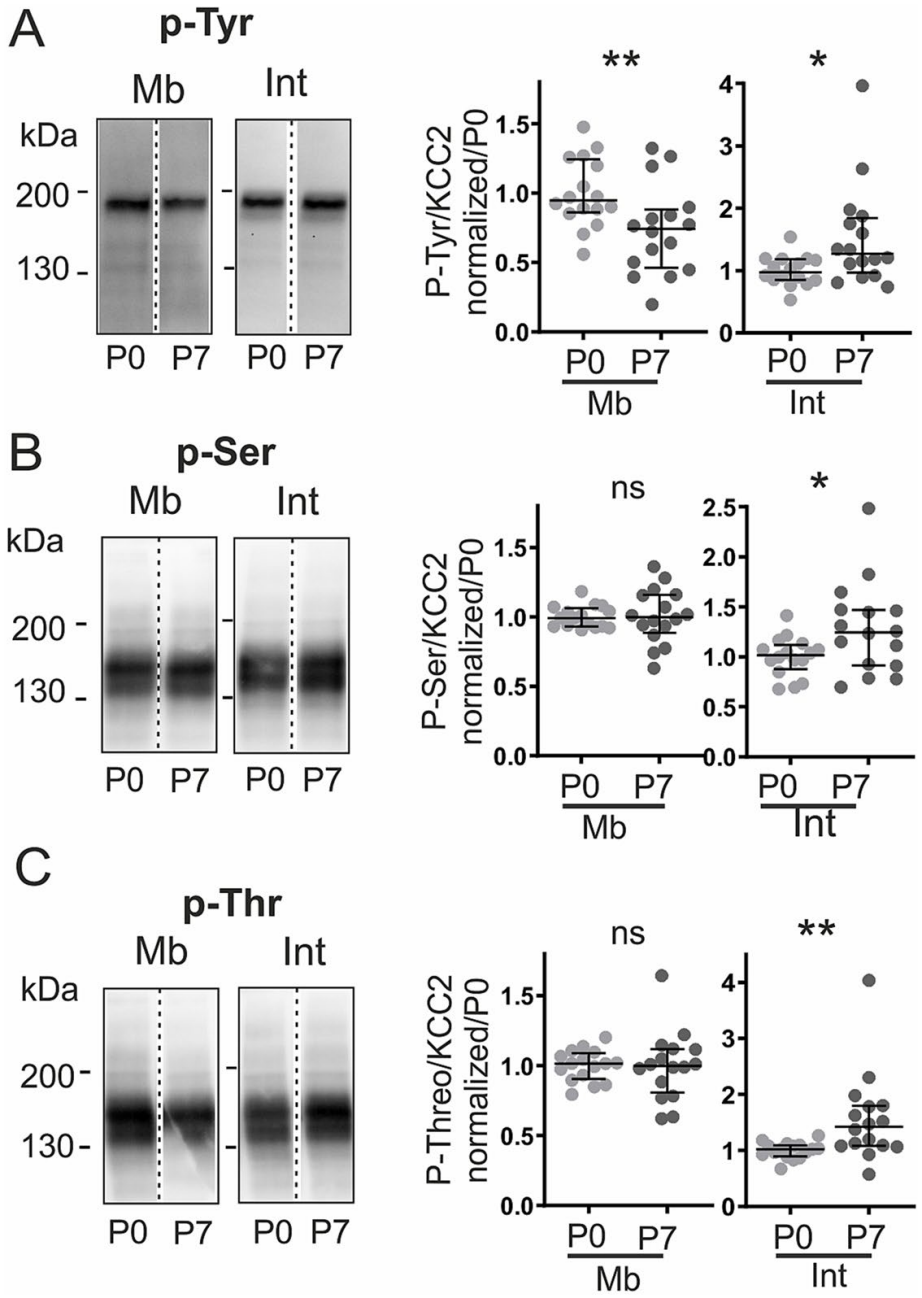
Developmental changes in KCC2 phosphorylation. Phosphorylation at P0 and P7. **(A–C)** Immunoblots and quantification for phospho-tyrosine (p-Tyr, **A**), phospho-serine (p-Ser, **B**), and phospho-threonine (p-Thr, **C**) in membrane (Mb) and intracellular (Int) fractions, normalized to monomeric KCC2 in each fraction. Values normalized to P0. Each dot consists of pooled spinal cords (four at P0, two at P7). *p < 0.05; **, *p <* 0.01; n = 16 per group; n represents pooled biological samples. Mann–Whitney test. Data are represented as median and interquartile range.

To determine whether the observed redistribution involved phosphorylation-dependent mechanisms, we examined KCC2 phosphorylation patterns at both developmental stages. Immunoprecipitation followed by phospho-specific immunoblotting revealed that only monomers were phosphorylated. Phosphorylation levels were therefore quantified as the ratio of phospho-signal to total monomeric KCC2 within each subcellular fraction. Intracellular monomers showed age-dependent increases in tyrosine (p-Tyr; +30%, *p <* 0.05), serine (p-Ser; +23%, *p <* 0.05), and threonine (p-Thr; +40%, *p <* 0.01) phosphorylation (Figures 2A-c). In contrast, membrane phosphorylation remained largely unchanged, except for tyrosine, which decreased by 26% (*p <* 0.01; Figure 2A). In addition, a distinct ~180 kDa band was detected in the tyrosine-phosphorylated fraction. Careful examination of whole lysates revealed that a weak but constant band was also observed at ~180 kDa by the anti-KCC2 antibody in all conditions (Figures 1A–C, Supplementary Figure S1a, arrow), indicative of a minor post-translationally modified KCC2 species. To understand the nature of this high molecular weight tyrosine-phosphorylated species (~180 kDa), we explored potential post-translational modifications beyond phosphorylation. As a preliminary investigation, we tested for SUMOylation. Western blot analysis revealed that the ~180 kDa species recognized by the anti-phosphotyrosine antibody was also immunoreactive to anti-SUMO1 in immunoprecipitated samples (Supplementary Figure S1d). These results indicate that the upper band of high molecular weight corresponds to a SUMOylated form of KCC2.

**Figure 3:**
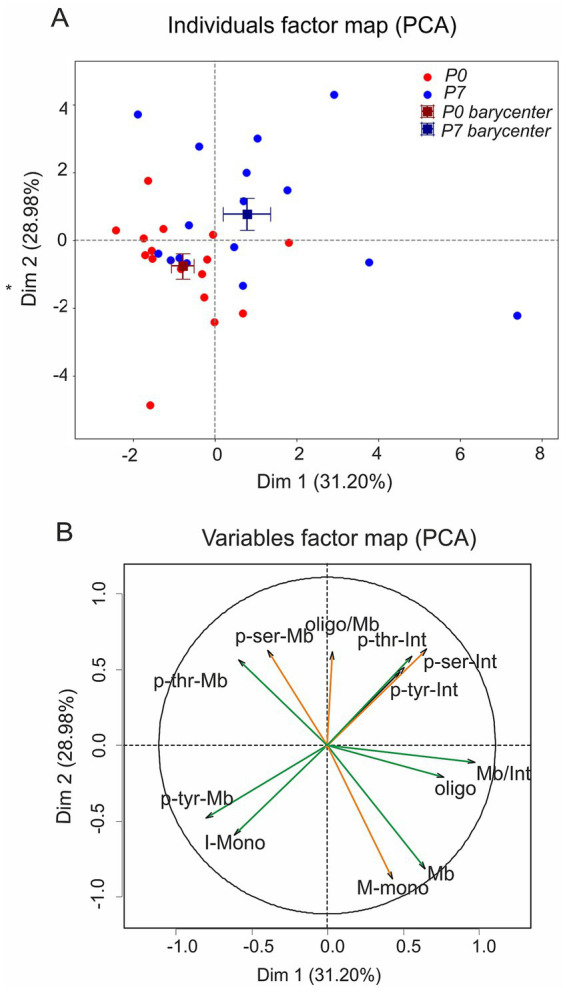
Principal component analysis of developmental KCC2 profiles. PCA of KCC2 expression from P0 to P7. **(A)** Individual factor map on Dim1 (31.2%) and Dim2 (28.98%), with P0 (red) and P7 (blue) samples, barycenters (squares), and standard error bars. Dim1 captures the developmental shift toward membrane localization, oligomerization, and increased intracellular phosphorylation, while Dim2 reflects variation in membrane phosphorylation levels. **(B)** Variable contributions: phosphorylation at tyrosine (p-Tyr), serine (p-Ser), and threonine (p-Thr) in membrane (Mb) and intracellular (Int) fractions, plus monomers (Mb-Mono), oligomers (Oligo), and oligomer-to-membrane ratio (Oligo/Mb). Each dot in **a** consists of pooled spinal cords (four at P0, two at P7).

Having established redistribution and phosphorylation dynamics at the molecular level, we next asked how these parameters cluster when considered together. Principal component analysis (PCA) identified two main dimensions accounting for 60% of the variance (Dim1: 31%, Dim2: 29%). P0 and P7 were clearly separated along Dim1 (*p <* 0.05; Figure 3A, Table 1), with P0 scoring negatively and P7 positively (V-test ± 2.3). As shown by a significant difference between barycenters, Dim1 drove this separation (*p <* 0.05; Table 1). Variables loading positively on Dim1 included a higher membrane-to-intracellular ratio (Mb/Int), greater oligomer abundance (Oligo), and elevated intracellular phosphorylation at serine, threonine, and tyrosine residues (p-Ser, p-Thr, p-Tyr). In other words, P7 samples were defined by a redistribution of KCC2 from the cytoplasm to the membrane, a shift toward the oligomeric form, and increased phosphorylation of intracellular monomers.

**Table 1:** PCA coordinates groups and statistics (P0 vs. P7).

DIM 1 coordinates		DIM 2 coordinates	
P0	P7	P0	P7
−24.254	0.4792	0.3038	−0.2079
−0.1918	17.673	−0.5595	14.855
−0.3.221	10.363	−10.026	29.957
−0.2.628	29.151	−16.838	42.823
0.6.825	−0.8598	−21.474	−0.5182
−0.8.322	0.6844	−0.8453	−13.387
−12.621	−0.6.947	0.3.292	−0.6718
−1.715	−10.759	−0.4411	−0.5837
−0.0102	−13.899	−24.036	−0.3821
−0.0441	−0.6417	0.1563	0.4517
−15.526	−0.386	−0.3139	27.599
−15.198	−18.893	−0.5434	37.044
−15.756	74.001	−48.533	−2.22
−17.349	37.664	0.0597	−0.6395
−16.313	0.7025	17.605	11.467
18.151	0.7682	−0.0716	19.919
*p < 0.05		ns *p* = 0.0731	

Quality of representation (Cos2)				
	DIM 1		DIM 2	
	Cos^2^	V test	Cos^2^	V test
P0	0.4947	−2.2627	0.4694	−2.3274
P7	0.4947	2.2627	0.4694	2.3274

The coordinates, Cos^2^ values, and V-test values for P0 and P7 are presented for both Dim1 and Dim2. Cos^2^: Quality of representation on each dimension. V-test: Significance of group position.

Beyond this developmental separation, the PCA also revealed inverse associations between membrane expression and phosphorylation levels. Membrane monomers (Mb-Mono) showed a negative correlation with serine (pSer-Mb) and threonine (pThr-Mb) phosphorylation, suggesting that dephosphorylation helps stabilize monomers at the surface. Likewise, oligomer abundance (Oligo) was inversely related to tyrosine phosphorylation (pTyr-Mb), indicating that tyrosine dephosphorylation favors oligomer formation and maintenance. Pairwise correlations within each component supported these relationships (Table 2).

**Table 2:** Variable contributions to PCA dimensions (P0 vs. P7).

Correlation table: P0/P7		
Dim 1	Correlation	*p*-value
Mb/Int	0.8763	< 0,0001
Oligo	0.6918	< 0,0001
pSer-Int	0.5901	0.0004
Mb	0.5826	0.0005
pThr-Int	0.5027	0.0034
pTyr-Int	0.4312	0.0137
Mb-Mono	0.3869	0.0287
pSer-Mb	−0.3522	0.048
pThr-Mb	−0.5263	0.002
Int-Mono	−0.5518	0.0011
pTyr-Mb	−0.718	< 0,0001

Dim 2	Correlation	*p-*value
pSer-Int	0.5726	0.0006
pSer-Mb	0.5655	0.0007
Oligo/Mb	0.5546	0.001
pThr-Int	0.532	0.0017
pThr-Mb	0.5092	0.0029
pTyrI-Int	0.4368	0.0124
pTyr-Mb	−0.4315	0.0137
Int-Mono	−0.5317	0.0017
Mb	−0.7279	< 0,0001
Mb-Mono	−0.7914	< 0,0001

Correlation coefficients (r) and *p*-values for each variable with Dim 1 (31.2%) and Dim 2 (29.0%).

Together, these findings indicate that postnatal development involves a coordinated redistribution of KCC2 from the cytoplasm to the membrane, accompanied by selective shifts in phosphorylation state. In particular, tyrosine dephosphorylation was consistently associated with the transition toward oligomerization, supporting a model in which phosphorylation state influences KCC2 stability during maturation.

### SCI reduces KCC2 oligomers and alters phosphorylation

**Figure 4:**
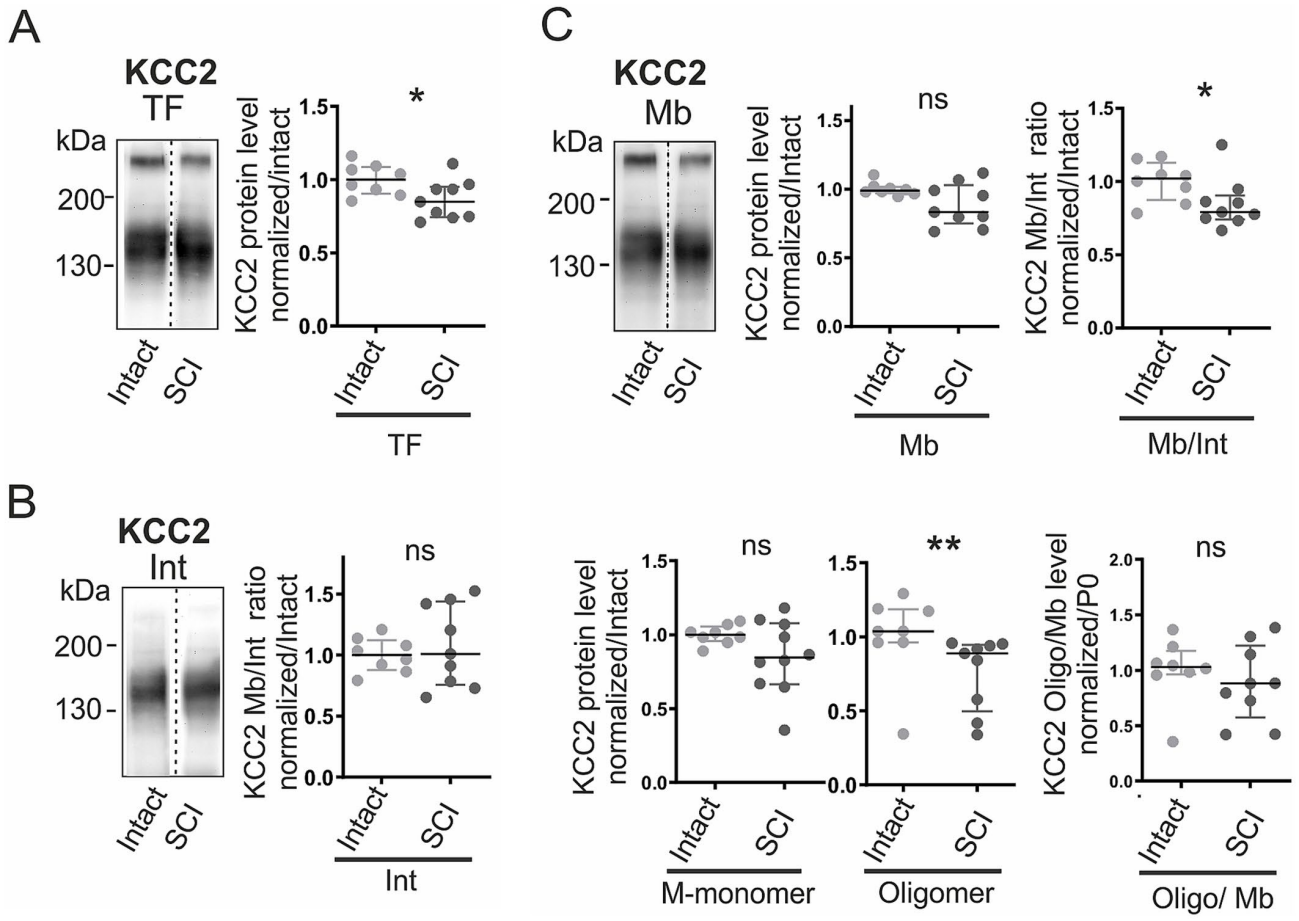
SCI reduces KCC2 membrane expression and oligomerization. KCC2 expression in intact vs. SCI rats at P7. **(A)** Total KCC2. **(B)** Intracellular fraction. **(C)** Membrane fraction: total (*top middle*), membrane-to-intracellular ratio (Mb/Int, *top right*), monomers (*bottom left*), oligomers (*bottom middle*), and oligomer-to-membrane ratio (*bottom right*). Values normalized to intact. Each dot consists of two pooled spinal cords. *, *p <* 0.05; **, *p <* 0.01; Intact P7, n = 8; P7 SCI, n = 9; FT P7 SCI, n = 10; n represents pooled biological samples. Mann–Whitney test. Data are represented as median and interquartile range.

When spinal cord injury (SCI) was performed at birth (P0), the normal postnatal upregulation of KCC2 was disrupted 1 week later. Total KCC2 levels were reduced by 15% (*p <* 0.05; Figure 4A). This decrease was not uniform across compartments: intracellular KCC2 remained unchanged (Figure 4B), whereas membrane expression declined, leading to a significantly lower Mb/Int ratio (*p <* 0.05; Figure 4c). The effect was mainly driven by a 15% loss of membrane oligomers (*p <* 0.01; Figure 4c), while membrane monomers were unaffected (ns; Figure 4c). Accordingly, the proportion of oligomeric KCC2 within the membrane pool was unchanged (Figure 4c).

**Figure 5:**
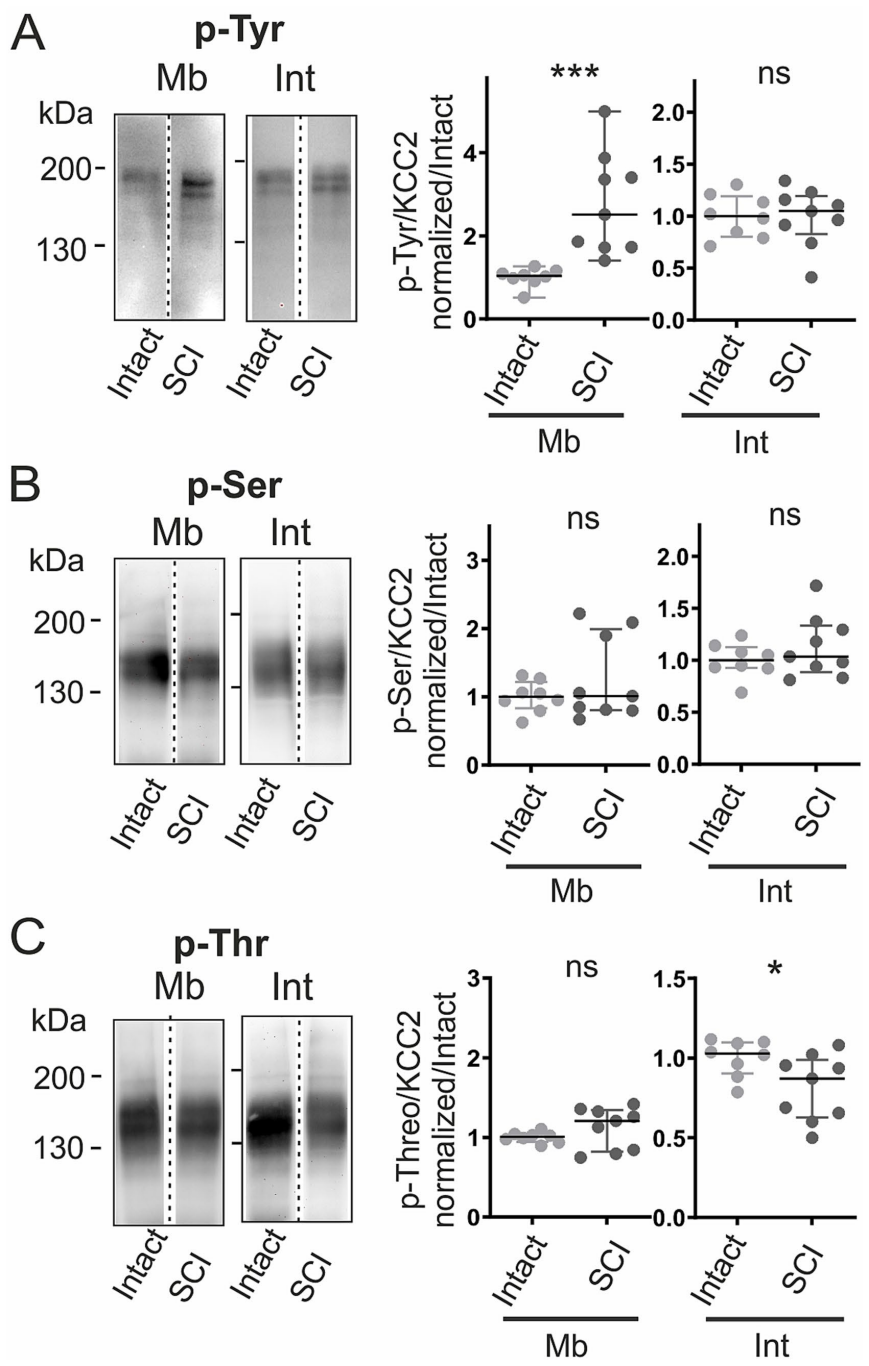
SCI alters KCC2 phosphorylation patterns. KCC2 phosphorylation in intact vs. SCI rats at P7. **(A–C)** KCC2 immunoblots of phosphorylated tyrosine (p-Tyr, **A)**, serine (p-Ser, **B)**, and threonine (p-Thr, **C)** in membrane (Mb) and intracellular (Int) fractions, normalized to monomeric KCC2 in each fraction. Values normalized to intact. Each dot consists of two pooled spinal cords. *, *p <* 0.05; **, *p <* 0.01; intact P7, *n* = 8; P7 SCI, *n* = 9; *n* represents pooled biological samples. Mann–Whitney test. Data are represented as median and interquartile range.

We next asked whether these expression changes were accompanied by modifications in the phosphorylation pattern. Membrane tyrosine phosphorylation increased sharply after SCI (+148%, *p <* 0.001; Figure 5A), reversing the normal developmental trend in which it declines (Figure 2A). In contrast, phosphorylation at serine and threonine residues remained unchanged at the membrane (Figs. 5B,c). Intracellularly, tyrosine and serine phosphorylation were unaffected (Figs. 5A,B), whereas threonine phosphorylation dropped by 16% (*p <* 0.05; Figure 5c).

**Figure 6:**
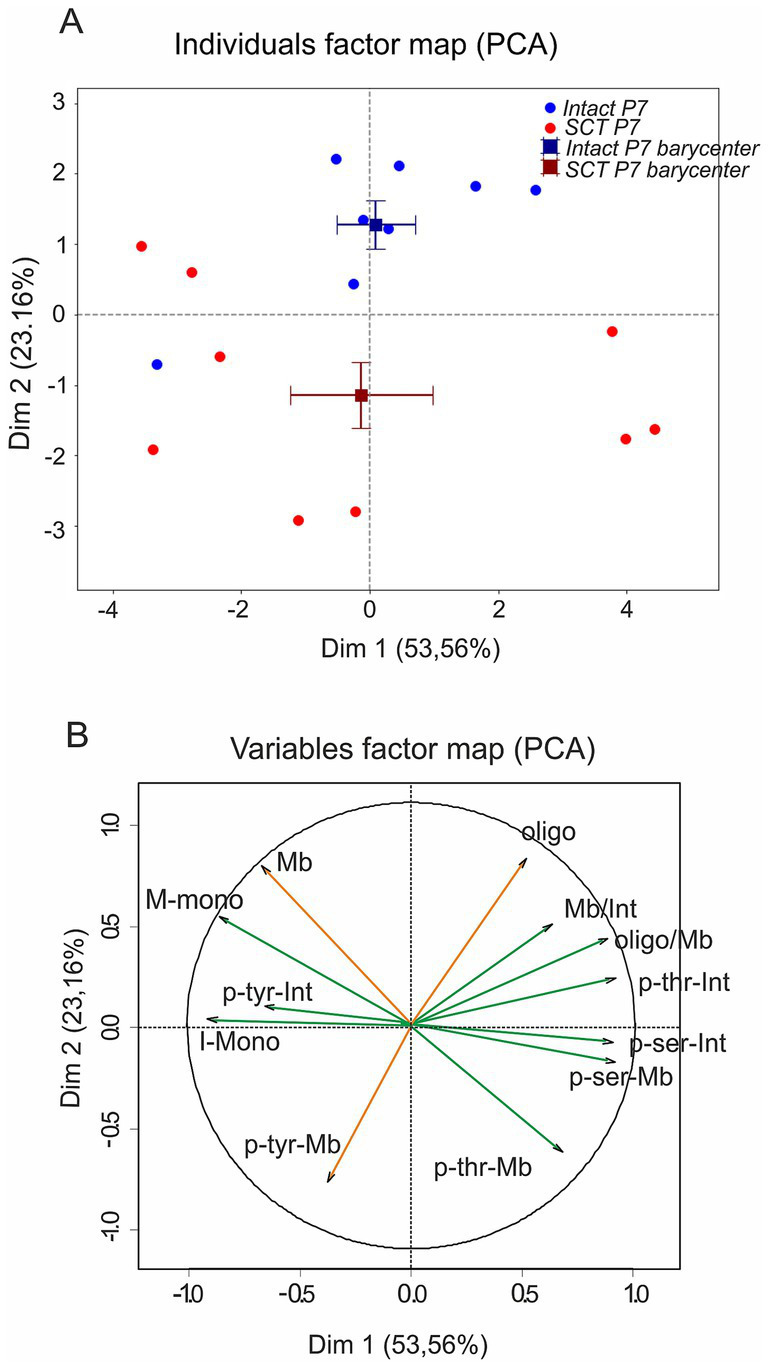
Principal component analysis reveals distinct KCC2 signatures in intact vs. SCI. PCA of KCC2 profiles at P7. **(A)** Individual factor map on Dim1 (53.56%) and Dim2 (23.16%), showing intact (blue) and SCI (red) samples with barycenters (squares) and standard error bars. Dim1 captures overall variation in KCC2 abundance and phosphorylation, while Dim2 discriminates intact from SCI samples based on oligomer content and membrane tyrosine phosphorylation. **(B)** Variables contributions: phosphorylation at tyrosine (p-Tyr), serine (p-Ser), and threonine (p-Thr) in membrane (Mb) and intracellular (Int) fractions, plus monomer (Mb-Mono), oligomer (Oligo), and oligomer-to-membrane ratio (Oligo/Mb). Each dot in **a** consists of two pooled spinal cords.

To integrate the expression and phosphorylation changes observed after SCI, we performed a PCA. Two components accounted for 77% of the variance (Dim1: 53.6%, Dim2: 23.2%; Figure 6A). Dim1 mainly captured overall variation in KCC2 abundance and phosphorylation but did not distinguish intact from SCI groups (V-test ≈ 0; Table 3). By contrast, Dim2 emerged as the discriminant axis, clearly separating the two conditions (V-test ±2.88; *p <* 0.01; Table 3). Variables loading positively on Dim2 included membrane localization and oligomer abundance, features that defined intact animals (Figure 6B; Table 4). SCI animals scored negatively on the same axis, characterized by reduced oligomers and increased phosphorylation at the membrane, especially tyrosine and threonine (pTyr-Mb, pThr-Mb; Figure 6B; Table 4).

**Table 3:** PCA coordinates groups and statistics (intact vs. SCI).

DIM 1 coordinates		DIM 2 coordinates	
Intact SCI	SCI P7	Intact SCI	SCI P7
−0.2596	0.2226	0.4406	−2.7967
0.4505	−1.1135	2.1164	−2.9089
−0.1005	−3.3759	1.3413	−1.9092
0.2913	−2.3446	1.2157	−0.5883
−3.316	−2.7775	−0.706	0.61
−0.5228	−3.5591	2.2184	0.9801
1.6375	4.4296	1.8316	−1.6193
2.583	3.9816	1.7701	−1.7599
	3.7734		−0.2359
ns		**p < 0.01	

Quality of representation (Cos2)				
	Dim 1		Dim 2	
	Cos^2^	V test	Cos^2^	V test
Intact P7	0.0046	0.1419	0.8254	2.8924
SCI P7	0.0046	−0.1419	0.8254	−2.8924

Coordinates, Cos^2^, and V-test values for intact P7 and SCI P7 on Dim1 and Dim2. Cos^2^: Quality of representation. V-test: Significance.

**Table 4:** Variable contributions to PCA dimensions (Intact vs. SCI).

Correlation table: P7/SCI		
DIM 1	Correlation	*p*-value
pSer-Mb	0.8536	< 0.0001
pSer-Int	0.8403	< 0.0001
Oligo/Mb	0.8097	0.0001
pThr-Int	0.799	0.0001
pThr-Mb	0.7057	0.0015
Mb/Int	0.6921	0.0021
Oligo	0.5028	0.0397
pTyr-Int	−0.5969	0.0114
Mb	−0.6628	0.0037
Mono	−0.8435	< 0.0001
Int	−0.9208	< 0.0001

**DIM 2**	**Correlation**	***p-*value**
Oligo	0.781	0.0002
Mb	0.7285	0.0009
Mb/Int	0.5269	0.0298
Mono	0.5114	0.0359
pThr-Mb	−0.5651	0.0181
pTyr-Mb	−0.702	0.0017

Correlation coefficients (r) and p-values for each variable with Dim1 (53.6%) and Dim2 (23.2%).

Together, these results indicate that SCI destabilizes KCC2 by reducing its oligomeric form and favoring a hyperphosphorylated, less stable state at the plasma membrane. This pattern contrasts with the normal postnatal development, where tyrosine dephosphorylation accompanies oligomerization and stabilization.

### DOI restores KCC2 expression and phosphorylation post-SCI

**Figure 7:**
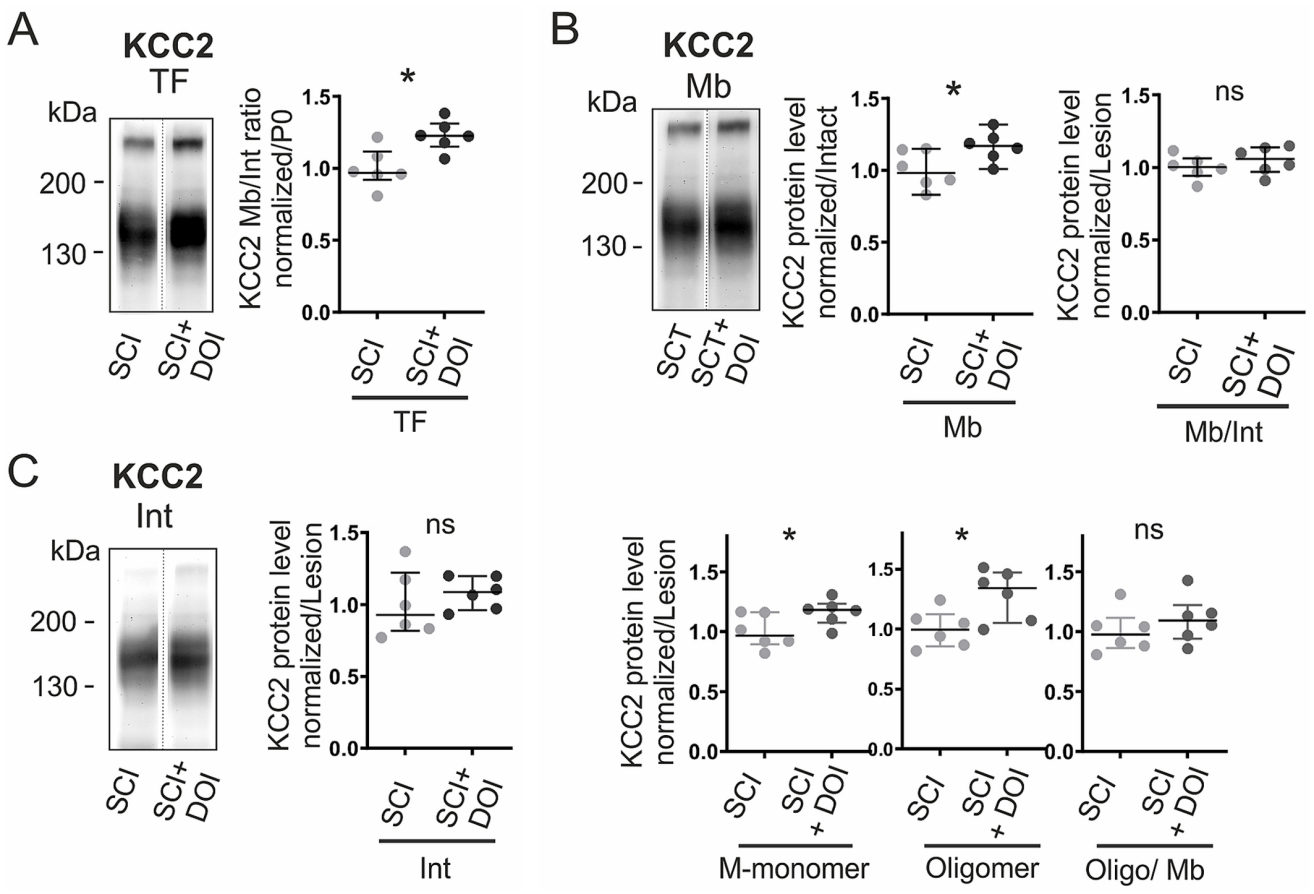
DOI restores membrane KCC2 expression after SCI. KCC2 in vehicle- vs. DOI-treated SCI rats at P7: **(A)** Total KCC2, **(B)** membrane fraction: total (*top middle*), membrane-to-intracellular ratio (Mb/Int, *top right*), monomers (Mb-Mono; *bottom left*), and oligomers (*bottom middle*) and the oligomer-to-membrane ratio (Oligo/Mb; *bottom right*), **(C)** intracellular fraction. Values normalized to vehicle. Each dot consists of two pooled spinal cords. *, *p <* 0.05; n = 6 per group; n represents pooled biological samples. Mann–Whitney test. Data are represented as median and interquartile range.

Building on our earlier work showing that activation of 5-HT_2_A/_2_C with DOI restores KCC2 expression after neonatal SCI (Bos et al., 2013), we used this condition to assess how recovery of expression relates to specific phosphorylation changes. One week after treatment, total KCC2 levels increased by 26% compared to SCI animals (*p <* 0.05; Figure 7A). This effect was driven by enhanced membrane expression (Figure 7B), with significant gains in both oligomers (+35%; *p <* 0.05) and monomers (+21%; *p <* 0.05). By contrast, intracellular levels remained unchanged (Figure 7c), and neither the membrane/intracellular distribution nor the oligomeric fraction of membrane KCC2 was significantly affected (Figure 7B). Thus, DOI boosts the total and membrane KCC2 amount after SCI, primarily by restoring both monomeric and oligomeric forms without altering their relative distribution.

**Figure 8:**
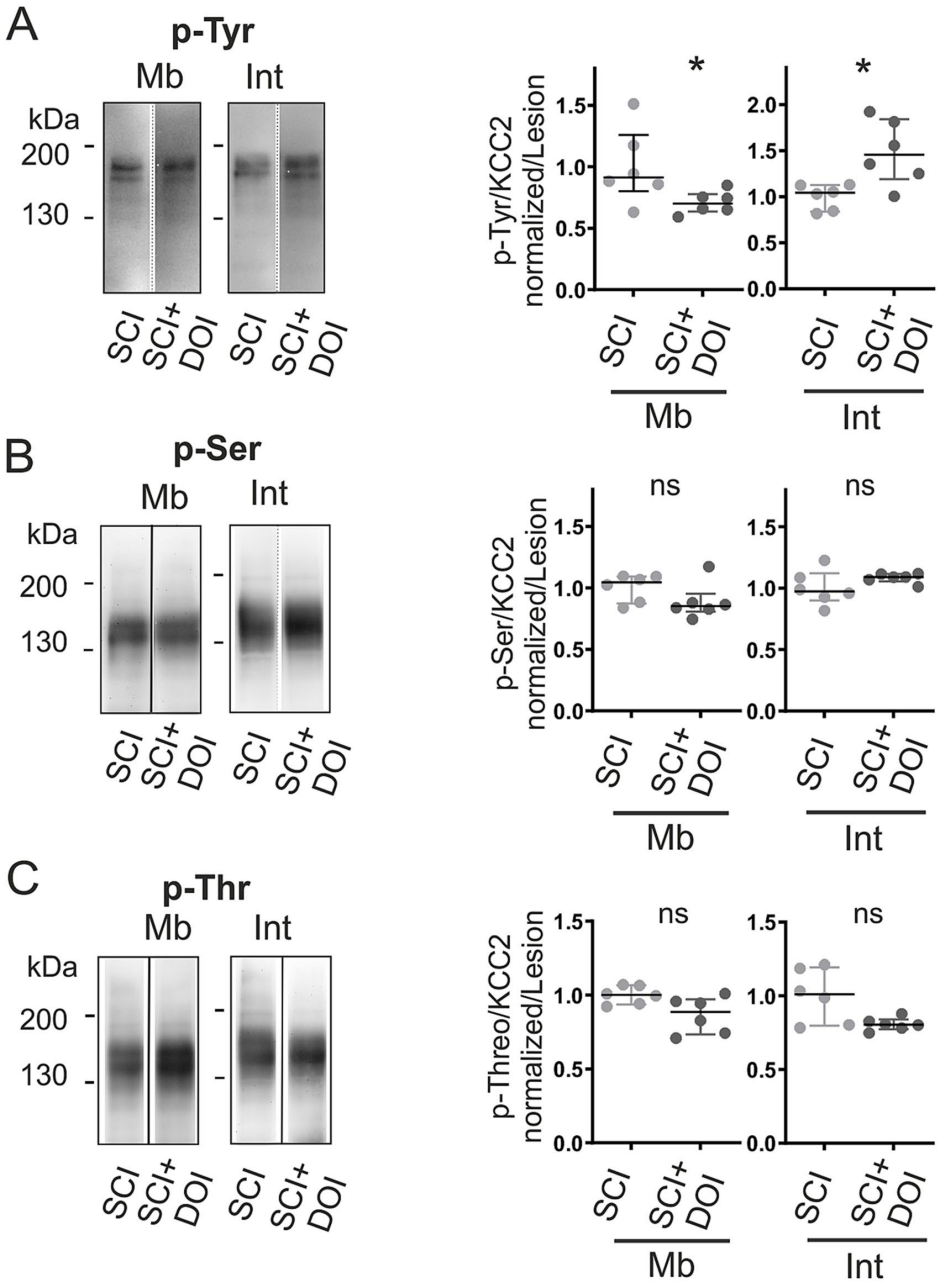
DOI normalizes KCC2 phosphorylation after SCI. Phosphorylation in vehicle- vs. DOI-treated SCI rats at P7. **(A–C)** KCC2 immunoblots of phosphorylated tyrosine (p-Tyr, **A)**, serine (p-Ser, **B)**, and threonine (p-Thr, **C)** in membrane (Mb) and intracellular (Int) fractions, normalized to monomeric KCC2 in each fraction. Values normalized to vehicle. Each dot consists of two pooled spinal cords.*, *p <* 0.05; *n = 6 per group; n* represents pooled biological samples. Mann–Whitney test. Data are represented as median and interquartile range.

In parallel with the expression recovery, we examined whether DOI also recovered the phosphorylation profiles to levels observed during developmental maturation. DOI treatment counteracted the SCI-induced hyperphosphorylation at the membrane, reducing tyrosine phosphorylation by 21% (*p <* 0.05; Figure 8A). At the same time, it enhanced tyrosine phosphorylation of intracellular monomers (+41%, *p <* 0.05; Figure 8A), a distribution that resembles the postnatal maturation. By contrast, phosphorylation at serine and threonine residues remained unchanged in both membrane and intracellular fractions (Figs. 8B,c), indicating that DOI specifically targeted tyrosine sites.

**Figure 9:**
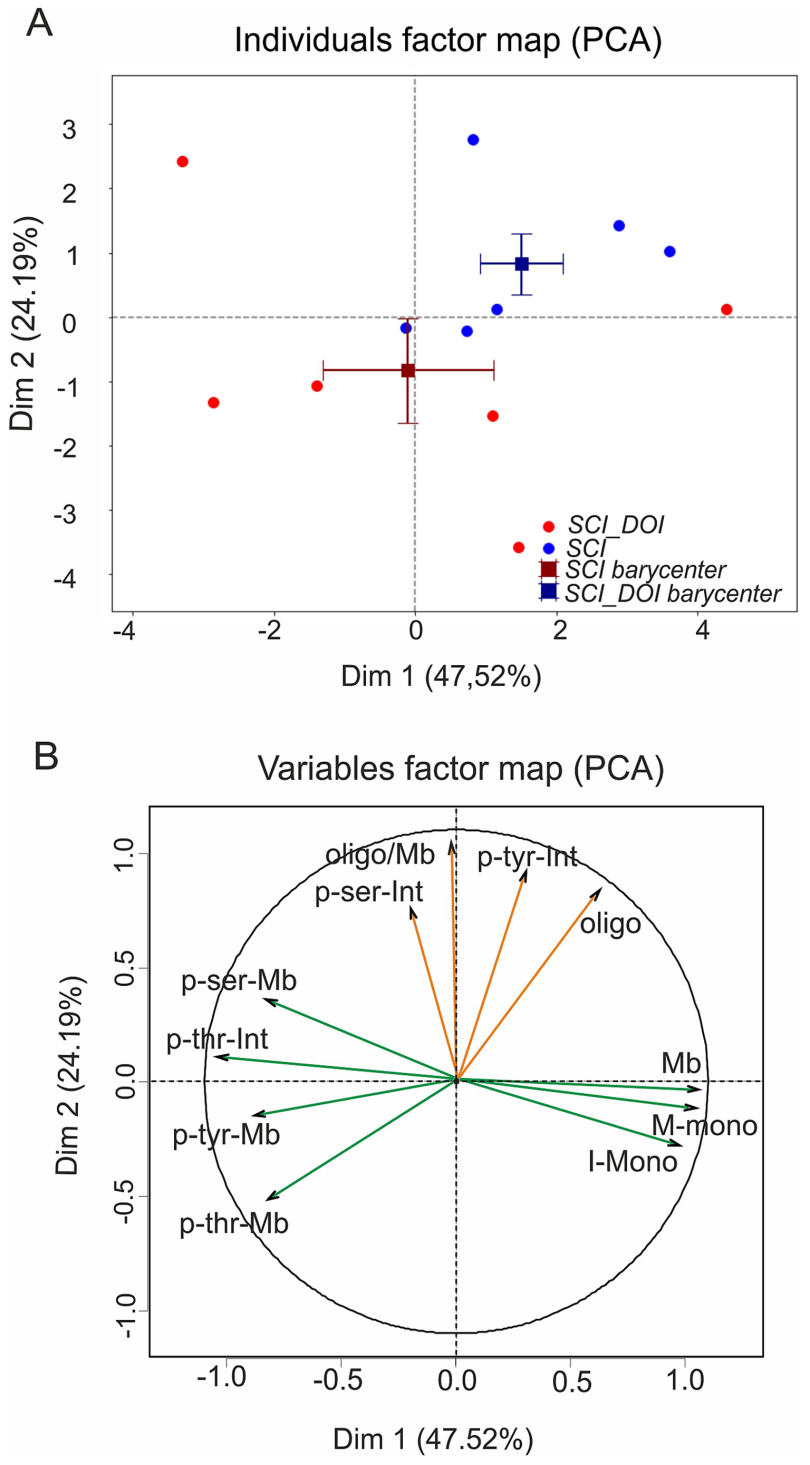
Principal component analysis shows DOI restores KCC2 toward a developmental-like state. Individual factor map on Dim1 (47.52%) and Dim2 (24.19%), with vehicle (blue) and DOI (red) SCI samples, barycenters, (squares) and standard error bars. Dim1 reflects the balance between membrane expression and phosphorylation levels, while Dim2 captures oligomer abundance and intracellular phosphorylation, along which DOI-treated animals separate from SCI controls. **(B)** Variables contributions: phosphorylation at tyrosine (p-Tyr), serine (p-Ser), and threonine (p-Thr) in membrane (Mb) and intracellular (Int) fractions, plus monomer (Mb-Mono), oligomer (Oligo), and oligomer-to-membrane ratio (Oligo/Mb). Each dot in **(A)** consists of two pooled spinal cords.

To integrate expression and phosphorylation profiles after DOI treatment, we performed a PCA. The first two components accounted for 72% of the variance (Dim1: 48%, Dim2: 24%; Figure 9A). Dim1 explained most of the variance (Cos2 = 0.68, V-test = ±2.03) but did not separate groups (Figure 9A; Table 5). Instead, Dim2 (Cos2 = 0.23, V-test = ±1.64) clearly distinguished SCI from DOI-treated animals, despite its lower representation quality (Figure 9A; Table 5). Variables contributing most strongly to Dim1 were overall membrane expression (Mb, Int-Mono) with negative loadings from phosphorylation variables (pTyr-Mb, pThr-Mb, pSer-Mb, pThr-Int; Figure 9B; Table 6), indicating that Dim1 mainly captured a balance between expression and phosphorylation. By contrast, Dim2 loaded on oligomer abundance (Oligo, Oligo/Mb) and intracellular phosphorylation (pTyr-Int, pSer-Int; Figure 9B; Table 6), providing the axis along which DOI-treated animals separated from SCI.

**Table 5:** PCA coordinates groups and statistics (SCI vs. SCI+DOI).

DIM 1 coordinates		DIM 2 coordinates	
SCI P7	SCI DOI	SCI P7	SCI DOI
−3.2988	3.6057	2.4204	1.0315
−2.86	2.8805	−1.3317	1.4291
1.4588	0.8135	−3.5811	2.7643
−4.4095	1.5027	0.1241	0.1295
−1.3958	0.7302	−1.0743	−0.2041
1.1053	−0.1326	−1.5422	−0.1654
**ns**		* *p <* 0.05	

Quality of representation (Cos2)				
	DIM 1		DIM 2	
	Cos^2^	V test	Cos^2^	V test
SCI P7	0.7325	−2.1736	0.2471	−1.6172
SCI DOI	0.7325	2.1736	0.2471	1.6172

Coordinates, Cos^2^, and V-test values for SCI P7 and SCI + DOI on Dim1 and Dim2. Cos^2^: Quality of representation. V-test: Significance (|V-test| > 2, *p <* 0.05).

**Table 6:** Variable contributions to PCA dimensions (SCI vs. SCI+DOI).

Correlation table: SCI/SCI+DOI		
DIM 1	Correlation	*p*-value
Mb	0.942	< 0.0001
Mono	0.9268	< 0.0001
Int-Mono	0.8387	0.0007
pSer-Mb	−0.7241	0.0077
pThr-Mb	−0.7849	0.0025
pTyr-Mb	−0.8147	< 0.0012
pThr-Int	−0.9426	< 0.0001

DIM 2	Correlation	*p*-value
Oligo/Mb	0.9009	0.0001
pTyr-Int	0.7096	0.0055
Oligo	0.7134	0.0092
pSer-Int	0.7439	0.0097

Correlation coefficients (r) and *p*-values for each variable with Dim1 (47.5%) and Dim2 (24.2%).

In sum, DOI restored a developmental-like phosphorylation profile of KCC2, shifting tyrosine phosphorylation from the membrane to the intracellular compartment, associated with enhanced oligomerization and membrane stabilization.

## Discussion

Our study shows that KCC2 expression and phosphorylation are dynamically regulated during postnatal maturation and that SCI severely disrupts these processes. Although the present study is not specifically designed to directly re-test function, we discuss our data in relation with previous evidence that causally links (i) KCC2 upregulation with functional maturation of inhibition (i.e., the shift of E*_Cl_*/E*_IPSP_* toward hyperpolarized values as KCC2 rises) (Blaesse et al., 2006; Jean-Xavier et al., 2006; Watanabe and Fukuda, 2015) and (ii) SCI phenotypes, together with functional recovery upon KCC2 enhancement, including 5-HT_2A_/_2C_ dependent strategies such as DOI (Boulenguez et al., 2010; Bos et al., 2013; Liabeuf et al., 2017; Sanchez-Brualla et al., 2018). Here we provide a mapping of the relevant biochemical organization of KCC2 (its subcellular distribution, oligomeric state, and phosphorylation signatures) and then combine these parameters with PCA to describe the coordinated biochemical states at each stage of development, SCI, and serotonergic stimulation.

Before discussing the biological significance of these findings, a methodological consideration regarding the detection of KCC2 oligomers is warranted. The SDS-resistant behavior of the >250 kDa KCC2 species likely reflects a combination of intermolecular disulfide bonds and non-covalent interactions, as dimers persist even under reducing conditions (Agez et al., 2017). Furthermore, native dimers may undergo structural rearrangements into exceptionally stable conformations that resist dissociation even under strongly denaturing treatments (Medina et al., 2014). Given that our protocol included iodoacetamide to limit post-lysis disulfide exchange and samples were heated at 70 °C, we interpret this band as a detergent-resistant KCC2 assembly, while acknowledging that SDS-PAGE does not resolve native architecture.

With this consideration in mind, consistent with earlier studies (Blaesse et al., 2006; Jean-Xavier et al., 2006; Watanabe and Fukuda, 2015), our data indicate that KCC2 oligomerization parallels the maturation of inhibition. During normal development, we observed that oligomer formation correlates with increased phosphorylation of intracellular monomers, while tyrosine phosphorylation at the membrane declined. One possible interpretation is a two-phase mechanism in which phosphorylation is associated with recruitment into an intracellular/processing pool and membrane delivery followed by dephosphorylation that permissively accompanies oligomer enrichment and stabilization at the membrane. This interpretation aligns with a broader principle observed in other cation-chloride cotransporters, where dephosphorylation frequently triggers activation of transport activity across multiple cellular systems (Jennings and Schulz, 1991; Kaji and Tsukitani, 1991; Bize and Dunham, 1994; Bize et al., 2000).

The increase in intracellular tyrosine phosphorylation observed during development is consistent with age-related patterns reported in other regions of the CNS (Stein et al., 2004; Vale et al., 2005). One possible upstream regulator of this phosphorylation is neurotrophin signaling. Activation of the BDNF–TrkB receptor has been shown to increase KCC2 expression and to accelerate the developmental shift of GABAergic signaling from excitation to inhibition, whereas loss of TrkB function impairs KCC2 expression and weakens inhibitory tone (Aguado et al., 2003; Carmona et al., 2006). Whether BDNF–TrkB acts directly on KCC2 or indirectly through downstream kinases, this pathway may couple developmental signals to the phosphorylation-dependent trafficking of KCC2.

Seven days after the lesion, we observed a reversal of the developmental pattern with higher membrane tyrosine phosphorylation and fewer KCC2 oligomers, a profile associated with transporter destabilization and internalization (Lee et al., 2010; Chowdhury et al., 2013). KCC2 thus appears trapped in a hyperphosphorylated pool, which compromises chloride extrusion. Importantly, DOI reversed this shift, lowering membrane tyrosine phosphorylation and restoring surface oligomers. This coordinated recovery is consistent with a permissive role for tyrosine dephosphorylation in oligomerization and membrane stabilization.

Threonine phosphorylation showed a different developmental profile. During postnatal maturation, the amount of threonine-phosphorylated KCC2 at the membrane remained largely unchanged, which contrasts with the prevailing view that threonine dephosphorylation activates KCC2. Removal of phosphate groups at Thr-906 and Thr-1007 has been shown to enhance KCC2 transport activity (Rinehart et al., 2009; Pisella et al., 2019), without noticeably altering surface expression (Rinehart et al., 2009; Inoue et al., 2012; Moore et al., 2018). Conversely, phosphomimetic mutations at the two threonine residues, or phosphorylation mediated by WNK1–3 kinases, inhibit KCC2 function (Kahle et al., 2005; de Los Heros et al., 2006; Rinehart et al., 2011; Friedel et al., 2015; Watanabe et al., 2019). It is possible that the functional relevance of threonine dephosphorylation emerges at later stages of development, beyond the P0-P7 window examined here.

We also found that serine phosphorylation rose in the cytoplasm between P0 and P7, matching the increase in membrane KCC2. This pattern points to a role for serine phosphorylation in promoting KCC2 delivery to the membrane. Although our pan-phospho antibodies do not resolve individual residues, this profile aligns with the specific regulation of Ser-940. Consistent with this idea, phosphorylation of Ser-940 by PKC has been shown to promote surface expression (Lee et al., 2007). Because 5-HT_2_A receptors recruit PKC signaling (Bos et al., 2013) and serotonergic projections reach the lumbar spinal cord during early postnatal weeks (Tanaka et al., 1992), endogenous serotonin is likely to contribute to the developmental rise in membrane KCC2. This interpretation is supported by the observation that neonatal serotonin depletion heightens lumbar excitability (Pflieger et al., 2002) and that DOI restores KCC2 function after SCI.

In addition to phosphorylation, KCC2 is influenced by other post-translational mechanisms. One example is calpain, a Ca^2+^-dependent protease that, when activated by excessive calcium influx, targets KCC2, causing its cleavage and functional loss (Puskarjov et al., 2012; Zhou et al., 2012; Chamma et al., 2013; Silayeva et al., 2015; Plantier et al., 2019). The C-terminal domain of KCC2 contains two PEST motifs that are calpain recognition sites, and their deletion abolishes both chloride extrusion and oligomerization, resulting in disinhibition (Mercado et al., 2006; Acton et al., 2012). Following SCI, oligomeric KCC2 was selectively reduced, while phosphorylated monomers in the cytoplasm remained stable. This suggests that tyrosine phosphorylation may spatially mask the cleavage sites or induce a conformation resistant to proteolysis, whereas dephosphorylated oligomers may constitute a particularly vulnerable pool for calpain-mediated breakdown. Supporting this interpretation, inhibition of the protease after SCI preserved oligomers (Plantier et al., 2019). Ser940 dephosphorylation following trauma may provide an additional destabilizing influence, promoting oligomer dissociation and worsening chloride dysregulation (Modol et al., 2014; Toda et al., 2014).

Although the variations in the oligomeric pool appear modest in absolute values (10–15%), previous functional studies showed that similar variations in the content of membrane KCC2 can have a major impact on the efficacy of inhibition. For example, a 12% decrease in spinal KCC2 after SCI was associated with a large depolarization of E*_Cl_* in motoneurons and spasticity-related outcomes (Boulenguez et al., 2010). In contrast, the same level of KCC2 rescue (11–16%) achieved by BDNF, 5-HT_2A_ receptor activation, prochlorperazine, exercise training, or calpain inhibition among others, has been found to result in significant functional improvements after SCI (Boulenguez et al., 2010; Bos et al., 2013; Cote et al., 2014; Liabeuf et al., 2017; Plantier et al., 2019; Kerzonkuf et al., 2024). Collectively, these results imply that small differences in the membrane fraction of KCC2, and perhaps in the proportion of KCC2 incorporated in stable oligomeric complexes, could be relevant from a physiological standpoint to fine-tune chloride homeostasis and inhibition.

During our analysis of tyrosine-phosphorylated KCC2, we observed an unexpected ~180 kDa band that prompted us to investigate possible SUMOylation. Our findings indicate that tyrosine-phosphorylated KCC2 can undergo SUMO conjugation, a modification that, to our knowledge, has not been previously reported for this transporter. Although in silico predictions identified two potential SUMOylation sites (FKAE and MKPE; Supplementary Table S1), we did not detect SUMO-conjugated oligomers in our biochemical fractions. While the functional relevance remains unclear, the known interplay between phosphorylation and SUMOylation in other transporters (Yao et al., 2011; Yao et al., 2014) warrants future investigation to determine whether such crosstalk modulates KCC2 trafficking and membrane stability.

Altogether, these results support a model in which phosphorylation acts as a modulator of KCC2 stability. Tyrosine dephosphorylation coincided with oligomer formation, consistent with a role in facilitating effective chloride extrusion. By contrast, phosphorylation of intracellular residues, probably via PKC, appeared to be associated with membrane trafficking, suggesting a role in transporter delivery rather than activation per se. After SCI, this coordination was disrupted, with increased membrane tyrosine phosphorylation and a selective loss of oligomers, consistent with a less stable transporter. The relationships we observe are correlative, yet they converge on a coherent picture of how phosphorylation dynamics may influence KCC2 stability. Taken together with the causal evidence from prior functional studies, modulating tyrosine dephosphorylation represents a potential avenue for recovering inhibition following SCI.

The inability to measure KCC2 phosphorylation in specific cell types represents a limitation of the current study as our biochemical analysis was performed on bulk lumbar spinal cord tissue. Although prior studies established the functional impact of KCC2 regulation specifically in motoneurons during this developmental window and after SCI (Jean-Xavier et al., 2006; Boulenguez et al., 2010; Bos et al., 2013), the role of interneurons and other cell types in the observed biochemical changes is currently unknown. Finally, the fact that our analyses are limited to an early neonatal transection model raises the question of whether the observed phosphorylation/oligomerization signatures might be the same in adult and/or chronic SCI. Future research using cell-type- specific methodologies, such as reporter or mouse lines for KCC2 or Cre-dependent lines, fluorescence-activated cell sorting combined with phospho-proteomics, or high-resolution phospho-specific imaging will be needed to determine the role of phosphorylation in the regulation of KCC2 in different populations of cells in the spinal cord. This would also allow to understand if the phosphorylation events we describe are uniform within cell types or represent population-specific regulatory mechanisms.

## Data Availability

The original contributions presented in the study are included in the article/Supplementary material, further inquiries can be directed to the corresponding authors.
