# Supplementary figures and images for: KCC2 phosphorylation dynamics relate to oligomerization during development and after spinal cord injury
Source: Front Mol Neurosci. 2026 Feb 4;19:1745037. doi: 10.3389/fnmol.2026.1745037 (PMC12913098; doi:10.3389/fnmol.2026.1745037)

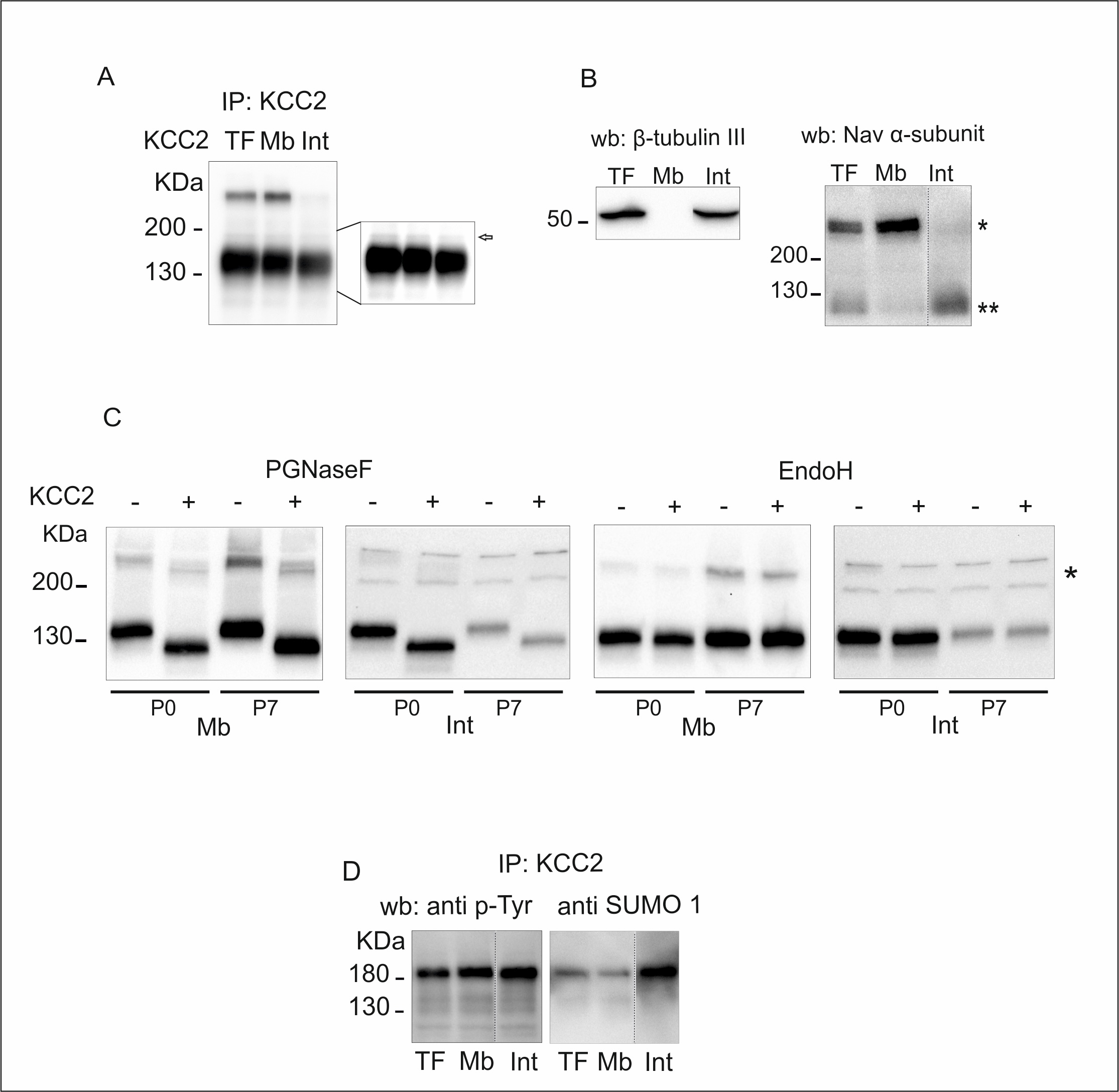

Supplement: Supplementary file 1 [file Image_1.jpeg]
